# A Regionalized Genome-Based Mexican Diet Improves Anthropometric and Metabolic Parameters in Subjects at Risk for Obesity-Related Chronic Diseases

**DOI:** 10.3390/nu12030645

**Published:** 2020-02-28

**Authors:** Claudia Ojeda-Granados, Arturo Panduro, Ingrid Rivera-Iñiguez, Maricruz Sepúlveda-Villegas, Sonia Roman

**Affiliations:** 1 Department of Molecular Biology in Medicine, Civil Hospital of Guadalajara “Fray Antonio Alcalde,” Hospital #278, Col. El Retiro, Guadalajara 44280, Mexico; claudiaojedagranados@hotmail.com (C.O.-G.); apanduro@prodigy.net.mx (A.P.); ingrid_rivei@hotmail.com (I.R.-I.); m_sep03@hotmail.com (M.S.-V.); 2 Health Sciences Center, University of Guadalajara, Guadalajara 44340, Mexico

**Keywords:** nutrigenetics, genome-based nutrition, traditional Mexican diet, staple foods, insulin resistance, lipids, adaptive genes, overweight, obesity, metabolic diseases

## Abstract

Obesity-related chronic diseases (CD) are highly prevalent in Mexicans who show moderate to high frequencies of diet-related adaptive gene (DRAG) polymorphisms and recent shifts in traditional dietary habits and lifestyles. This study first evaluated the effects of a regionalized genome-based Mexican (GENOMEX) diet on anthropometric and biochemical parameters and, subsequently their relationship with the genetic profile of DRAG polymorphisms in subjects with metabolic risk factors for obesity-related CD. Thirty-seven eligible subjects underwent a 24-week dietary intervention with a GENOMEX diet. The DRAG polymorphisms were determined by an allelic discrimination real-time assay to evaluate their association with the clinical response to diet. The GENOMEX diet significantly improved anthropometric parameters such as total weight, body mass index, waist circumference, and body fat percentage, with an average weight loss of 6.6% (5.3 ± 5.3 kg). The frequency of subjects with insulin resistance, hypertriglyceridemia and elevated VLDL-c (48.5% vs. 24.2%, *p* = 0.041; 45.5% vs. 12.1%, *p* = 0.003; and 39.4% vs. 15.2%, *p* = 0.027, baseline vs. 24-weeks, respectively) was reduced. A more significant favorable effect in HOMA-IR and insulin was observed in *MTHFR* 677T adaptive allele carriers, but no other DRAG polymorphism was associated with clinical changes. The GENOMEX diet improved the metabolic risk factors for obesity-related CD. The recommendation and habitual consumption of a traditional Mexican diet based on knowledge of the population’s genetic and cultural history may be effective in preventing current obesity-related CD.

## 1. Introduction

The present-day obesity pandemic and incidence of obesity-related chronic diseases (CD) are increasing in parallel with dramatic changes in traditional lifestyles and dietary habits of populations worldwide [1]. The physiopathological mechanisms underlying the metabolic diseases common in modern populations may rely on their particular evolutionary history that currently influences their patterns of disease risk [2]. Accordingly, populations presenting diet-related adaptive gene (DRAG) polymorphisms selected by their ancestral dietary contexts and currently exposed to the well-described Western dietary pattern and lifestyle are at increased risk of abnormal metabolic conditions and obesity-related CD [3,4,5]. Not more than 500 years ago the Mexican Native American (NA) populations, who relied on a pre-Hispanic diet since the emergence of plant domestication and agriculture (at least 5 kya), were subjected to a genetic and food-culture admixture with the arrival of Spaniards and the introduction of new ingredients from the Old World [6].

Today, obesity, type 2 diabetes mellitus (T2DM), cardiovascular diseases, dyslipidemias, and non-alcoholic fatty liver disease are highly prevalent in Mexicans related to a nutrition transition, which is shifting the traditional consumption of the ancestral pre-Hispanic diet based on the staple Mesoamerican foods fused with some healthy European ingredients towards an unhealthy hepatopathogenic diet [7,8]. Furthermore, a preceding analysis of DRAG polymorphisms in Mexican populations showed a high prevalence of adaptive alleles (methylenetetrahydrofolate reductase-*MTHFR* 677T, ATP-binding cassette transporter A1-*ABCA1* 230C, apolipoprotein E -*APOE ε4*, and a salivary amylase 1-*AMY1* diploid copy number ≥ 6) mainly in NA groups followed by admixed populations (Mestizo) with an intermediate NA ancestry, while a very low frequency of the European lactase-persistence *LCT*-13910T adaptive allele was observed [9]. These findings suggest that carriers of the adaptive alleles might require nutritional needs concordant with the pre-Hispanic diet and practices to prevent current disease-related risks [10,11,12,13,14,15,16]. In line with this hypothesis is the fact that polymorphism-disease associations are not replicated when studying populations that follow their traditional diet and lifestyle [17,18]. Additionally, some studies indicate that when standard therapeutic dietary recommendations for weight loss or treatment of obesity-related CD are ineffective, a personalized or regionalized nutritional approach is required, in which the genetic diversity and cultural context may determine individuals’ responsiveness and compliance to specific dietary interventions [19,20,21]. Herein, the rationale in accordance with the adaptive alleles and their observed prevalence, particularly in the Mexican Mestizo population of Western Mexico, was to design a regionalized genome-based Mexican (GENOMEX) diet to assure an adequate dietary folate intake (*MTHFR* 677T allele), a controlled ingestion of saturated fat/cholesterol-rich foods (*ABCA1* 230C and *APOE* ε4 alleles), the exclusion of milk (instead due to the high prevalence of the lactase non-persistence *LCT*-13910C allele) and the consumption of low-glycemic index starchy foods as a source of carbohydrates and fiber (*AMY1* diploid copy number of 6.82 ± 3.3) by including mainly regional Mexican foods that are known as staples of the pre-Hispanic nourishment [9,22].

Notably, compared to a large number of studies examining the benefits of traditional diets such as the widely recommended Mediterranean diet [23,24], there are very few studies evaluating the health effects of the traditional Mexican diet [25,26,27]. Therefore, this study aimed first to evaluate the effects of a regionalized GENOMEX diet on biochemical and anthropometric parameters and, subsequently, their relationship with the genetic profile of DRAG polymorphisms in subjects with metabolic risk factors for obesity-related CD.

## 2. Subjects and Methods

### 2.1. Study Participants

An invitation letter was distributed through leaflets to the general adult population. Participants were recruited at the Nutrigenetic Clinic, Department of Molecular Biology in Medicine, Civil Hospital of Guadalajara “Fray Antonio Alcalde,” Guadalajara, Mexico. Entry criteria included sedentary subjects aged 20–65 years, body mass index (BMI) ≥18 kg/m^2^, excess of adiposity determined by body fat percentage (BFP), and the presence of one or more of the following metabolic risk factors for obesity-related CD: serum triglycerides (TG) ≥ 150 mg/dL, total cholesterol (TC) ≥ 200 mg/dL, low-density lipoprotein cholesterol (LDL-c) ≥130 mg/dL, very low-density lipoprotein cholesterol (VLDL-c) ≥ 30 mg/dL, high-density lipoprotein cholesterol (HDL-c) ≤ 40 mg/dL and a homeostasis model assessment of insulin resistance (HOMA-IR) ≥ 2.5 [28,29]. After screening, subjects with T2DM, primary dyslipidemia, viral hepatitis, renal disease, or any other medical condition were excluded. Additionally, pregnant women and individuals habitually taking multivitamins, synthetic or herbal drugs, significant alcohol consumption (≥ 20 g/day and ≥ 40 g/day for women and men, respectively), smokers and those who were under any dietetic regimen or had any surgical procedure within the last three months were excluded. All subjects provided written informed consent for recruitment and at final enrollment. The present study was performed in accordance with the ethical principles for medical research in humans (Declaration of Helsinki 2013) and was approved by the Institutional Review Board of the Civil Hospital of Guadalajara (Ethic Approval Code 141-09).

### 2.2. Study Design

A quasi-experimental study with a one-group pretest-posttest design was performed in 37 eligible subjects (42.6 ± 11.7 years, 28 women and nine men) that underwent a 24-week dietary intervention with a regionalized GENOMEX diet. Anthropometric and biochemical parameters were measured at baseline and after 14 and 24 weeks. The genetic profile of certain DRAG polymorphisms were also determined in the subjects to evaluate subsequently their association with the response to diet.

### 2.3. Anthropometric Assessment

Anthropometric measurements were assessed using standard procedures with the subjects fasting (12 h) and wearing a disposable gown [30]. Standing height (to the nearest 0.1 cm) was measured using a stable stadiometer (seca GmbH & Co. KG, Hamburg, Germany) and waist circumference (to the nearest 0.1 cm) using a steel measuring tape (Rosscraft Innovations Inc., Vancouver, BC, Canada). The body composition parameters, including body weight, body water, protein mass, fat mass, and BFP were determined by bioelectrical impedance analysis using the InBody 3.0 analyzer (InBody Co., Seoul, Korea). BMI was calculated as body weight divided by height squared (kg/m^2^). Excess of adiposity was ascertained considering the BFP cut-off values by sex and age that have been associated with risk of metabolic syndrome (i.e., > 20%, > 22%, > 5% and > 28%, > 30%, > 32% in men and women in the 20–29, 40–49 and > 60-year age ranges, respectively). Normal weight, overweight, and obesity were determined according to such BFP classification [31,32]. A waist circumference ≥ 80 cm in women and ≥ 90 cm in men was considered of increased risk for obesity-related comorbidities [33].

### 2.4. Laboratory Tests

Venous blood samples were drawn after 12-h overnight fasting. Glucose, insulin, TC, TG, HDL-c, alanine aminotransferase (ALT), aspartate aminotransferase (AST) and gamma-glutamyl-transferase (GGT) concentrations were determined (AU5800 clinical chemistry analyzer, Beckman Coulter Inc., Brea, CA, USA). LDL-c was estimated with the Friedewald formula [34], the VLDL-c concentration as TC − (LDL-c + HDL-c), while HOMA-IR was calculated as [fasting plasma glucose (mg/dL) × fasting serum insulin (μU/mL)]/405 [35]. Insulin resistance (IR) was defined as HOMA-IR ≥ 2.5 [36].

### 2.5. Features of the Regionalized GENOMEX Diet

The design of the regionalized GENOMEX diet was in line with a previous proposal of our research group about providing intervention strategies considering the population’s genetic background and food culture for the prevention/management of obesity and related comorbidities [19,37]. It also considered the findings of the prevalence profile in Mexican populations of some DRAG polymorphisms that have been found with signatures of adaptive selection associated with ancestral dietary practices [9]. The adaptive alleles of these polymorphisms are related to diets rich in dietary folate (*MTHFR* 677T allele), low in saturated fat and cholesterol-rich foods (*ABCA1* 230C and *APOE* ε4 alleles), with dairy consumption (lactase persistence *LCT*-13910T allele), and abundance of starchy foods (*AMY1* diploid copy number ≥ 6). The regionalized GENOMEX diet pointed to assure an adequate dietary folate intake (> 300 µg/d), a controlled ingestion of saturated fat/cholesterol-rich foods, the exclusion of milk (due to the high prevalence of the lactase non-persistence *LCT*-13910C allele), and the consumption of low-glycemic index starchy foods as a source of carbohydrates (*AMY1* diploid copy number of 6.82 ± 3.3) and fiber [9]. Regional and seasonal Mexican foods that are known as staples of the pre-Hispanic nourishment (e.g., beans, tortilla, amaranth, avocado, chia seeds, pumpkin seeds, tomato, squash, different types of chilies, nopal, quelites, among others) were mainly included in the meal plans to meet these primary features. Meal plans were elaborated using the NutriKcal^®^ VO software (Consinfo, S.C., CDMX, Mexico) based on the Mexican System of Food Equivalents [38]. Twelve different 7-day meal plans of variable caloric content (1300–1600 kcal) were designed to be rotated throughout the dietary intervention. Detailed instructions and recipes were included for cooking each daily menu comprising three meals and two snacks. The mean values of the menus’ nutrient distribution are depicted in Table 1 [39].

### 2.6. Dietary Assessment

The subjects’ habitual energy and nutrient intake were evaluated at baseline with a 3-day dietary record using the NutriKal^®^ VO software. Subjects energy requirements were estimated considering their basal metabolic rate displayed by the InBody 3.0 analyzer, the degree of physical activity corresponding to a sedentary level (5–10%), and the percentage of the thermic effect of food (10%). Meal plans were assigned according to energy needs. Adherence (adequacy percentage) to the diet was evaluated at 14 weeks by a 24-h dietary recall and a food frequency questionnaire.

### 2.7. Genetic Analysis

DNA was extracted from leukocytes [40] and stored at −70 °C at a concentration of 20 ng/µL. DRAG polymorphisms were determined as detailed elsewhere [9]. Concisely, predesigned TaqMan^®^ SNP Genotyping Assays (Applied Biosystems, Life Technologies, Foster City, CA, USA) were used for the determination of polymorphisms *LCT*-13910 C>T (C__2104745_10), *MTHFR* 677 C>T (C__1202883_20), *ABCA1* Arg230Cys (C__11720861_10), and *APOE* T388C (C__3084793_20), C526T (C__904973_10) by a real-time PCR technique. The *AMY1* diploid gene copy number was examined by duplex quantitative real-time PCR using the TaqMan^®^ *AMY1* copy number assay (Hs07226362_cn; Applied Biosystems, Life Technologies) and TaqMan^®^ copy number reference assay for human RNase P (Applied Biosystems, Life Technologies).

### 2.8. Statistical Analysis

A sample size of 32 participants was estimated using the formula for quantitative data in a single study group, with a power of 95%, an α error of 0.05, and a maximum allowed sampling error of 0.04. It was calculated considering the standard deviation ± 11.49 mg/dL of the HDL-c mean value (42.13 mg/dL) previously reported for the adult Mexican population [41], which was one of the biochemical parameters expected to be modified by the dietary intervention. Thirty percent more participants were added due to possible losses during study follow-up, resulting in a sample size of 37 subjects. Continuous variables were expressed as mean ± standard deviation, while categorical variables as frequency and percentage. The Kolmogorov–Smirnov test with Lilliefors significance correction was used to assess the normality of continuous variables. A repeated-measures ANOVA was used to assess the dietary intervention effects on anthropometric and biochemical parameters, comparing the mean changes between baseline, 14- and 24-week measurements. Paired-samples *t*-tests were applied to evaluate and compare the mean changes of pre- to post-intervention of all the parameters and independent-samples *t*-tests to compare the baseline dietary features of study subjects with those of the GENOMEX diet. The equivalent Friedman and Wilcoxon tests were applied to analyze those variables with skewed distribution. The chi-square (χ^2^) test was used to compare the categorical variable of body composition as well as to test for the Hardy–Weinberg equilibrium (HWE). Mann–Whitney U test was applied to assess the association of the mean total changes in anthropometric and biochemical parameters for each DRAG polymorphism. For this purpose, the groups with and without the presence of each adaptive allele were tested. A two-tailed *p*-value < 0.05 was considered significant. All the statistical analyses were performed with the SPSS v.20 software.

## 3. Results

### 3.1. Baseline Characteristics

Thirty-seven subjects (42.6 ± 11.7 years, 28 women and 9 men) with metabolic risk factors for obesity-related CD were enrolled. The mean excess body weight (16.2 ± 10.1 kg) was entirely explained by the excess of fat mass (16.2 ± 10.1 kg). The mean excess fat mass was 15.6 ± 10.5 kg in women, and 17.9 ± 9.1 kg in men, and differences in the BFP between genders were found (37.6 ± 6.4% in women vs. 30.5 ± 5.7% in men, *p* = 0.005). According to the classification of body composition by sex- and age-specific BFP cut-off values, 21 (56.8%) subjects were overweight, and 16 (43.2%) were obese with a mean BMI of 26.4 ± 2.2 kg/m^2^ and 35.6 ± 3.8 kg/m^2^, respectively. The mean waist circumference of 96.6 ± 14.8 cm reflected an increased risk for obesity-related comorbidities, overall and by gender (94.1 ± 15.0 cm in women vs. 103.7 ± 12.4 cm in men, *p* = 0.094).

Their baseline dietary pattern was characterized by an excessive total energy, saturated fatty acids, and cholesterol intake, but inadequate polyunsaturated fatty acids, fiber, and folates intake (Table 1). The main metabolic risk factors were IR in 19 (51.4%) individuals, hypoalphalipoproteinemia in 18 (48.6%), elevated TG in 18 (48.6%), elevated VLDL-c in 16 (43.2%), increased TC in 14 (37.8%), increased LDL-c in 10 (27.0%), elevated GGT in 8 (21.6%), and elevated ALT in 7 (18.9%) subjects.

### 3.2. Genetic Profile of DRAG Polymorphisms

As shown in Table 2, a 75.7% of study subjects had the lactase non-persistence *LCT*-13910 CC genotype; 48.6% were *MTHFR* 677 CT or TT; 24.3% had an *ABCA1* 230 RC genotype, 9.5% were *APOE* ε4 allele carriers, and 51.4% had an *AMY1* diploid number of ≥ 6 copies. All biallelic DRAG polymorphisms complied with the HWE.

### 3.3. Metabolic and Anthropometric Response to the Regionalized GENOMEX Diet

Out of 37 study participants, 33 (42.6 ± 11.7 years, 25 women and eight men) completed the 24-week dietary intervention. Table 3 shows the dietary intervention effects on anthropometric and biochemical parameters in these subjects. There were significant favorable changes in all the anthropometric parameters, except in body water and muscle mass since the weight loss was only due to fat mass loss. Mean changes were only significant when baseline measurements were compared with 14-weeks and final measurements, as after 14-weeks, no significant anthropometric gains or losses were observed. Generally, subjects achieved an average loss of 6.6% (5.3 ± 5.3 kg) of their initial weight. Similarly, significant changes were observed in body composition classification, 12 (36.4%) individuals initially classified overweight achieved normal weight, and 2 (6.06%) subjects with obesity changed to overweight. However, 8 (24.2%) and 11 (33.3%) subjects remained in the overweight and obesity category, respectively. Participants also showed a significantly favorable change in waist circumference. By gender, women who began with a mean waist circumference of 92.9 ± 14.4 cm achieved 85.9 ± 13.6 cm, while men beginning with 102.8 ± 13.0 cm ended up with 96.0 ± 13.5 cm.

**Figure 1:**
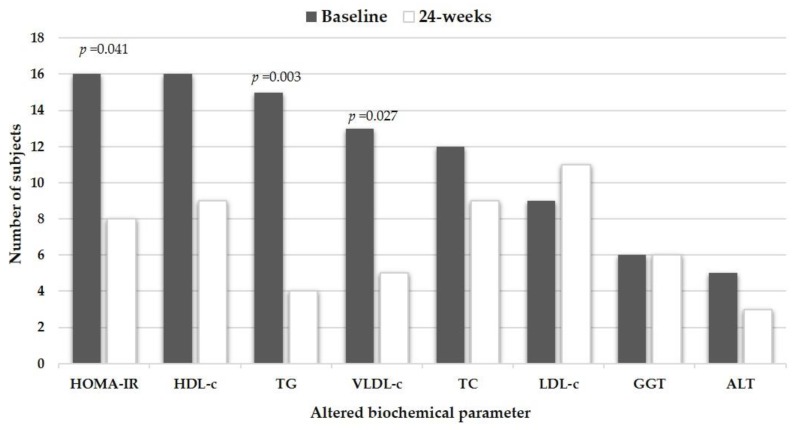
Metabolic disturbances in 33 study subjects before and after dietary intervention with a regionalized GENOMEX diet. Significant *p*-values are reported for the baseline vs. post-intervention change in the frequency of subjects with the altered metabolic parameter.

Regarding the biochemical parameters, the initial elevated mean values of HOMA-IR, TG, and VLDL-c significantly normalized at 14-weeks and were maintained until the end of the dietary intervention. Likewise, when comparing baseline vs. post-intervention state of the parameters, a significant final decrease in mean glucose and insulin values was observed, but LDL-c increased (Table 3). Figure 1 displays the frequency of individuals with metabolic risk factors at baseline and after nutritional treatment. The regionalized GENOMEX diet significantly reduced the frequency of subjects with IR (48.5% vs. 24.2%, *p* = 0.041), hypertriglyceridemia (45.5% vs. 12.1%, *p* = 0.003) and elevated VLDL-c (39.4% vs. 15.2%, *p* = 0.027) as well as an important proportion of participants normalized hypoalphalipoproteinemia (48.5% vs. 27.3%, *p* = 0.076).

### 3.4. Metabolic and Anthropometric Response to Diet in Relation to the Genetic Profile of DRAG Polymorphisms

**Figure 2:**
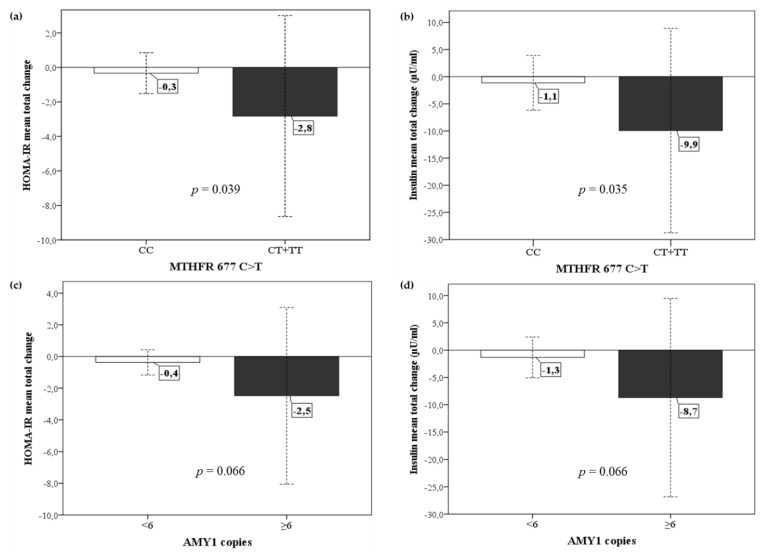
Mean changes in HOMA-IR and insulin after dietary intervention in relation to the DRAG polymorphisms in *MTHFR* and *AMY1*. (**a**) Comparison of mean total HOMA-IR change between *MTHFR* CC vs. CT+TT carriers. (**b**) Comparison of mean total insulin change between *MTHFR* CC vs. CT+TT carriers. (**c**) Comparison of mean total HOMA-IR change between subjects with < 6 vs. ≥ 6 copies of *AMY1*. (**d**) Comparison of mean total insulin change between subjects with < 6 vs. ≥ 6 copies of *AMY1*. Values are the mean ± standard deviation of the total changes (baseline vs. 24-weeks). *p*-values of the Mann–Whitney U test are reported.

The mean total changes achieved in the anthropometric and biochemical parameters after the dietary intervention were compared between carriers and non-carriers of each adaptive genetic variant. The mean decrease in HOMA-IR and insulin was significantly higher in subjects with the *MTHFR* 677 CT or TT genotypes than those with the CC genotype (Figure 2a,b). The same metabolic improvement was observed in subjects with ≥ 6 copies of *AMY1* compared with those with < 6 copies, although this change did not reach statistical significance (Figure 2c,d). No other adaptive allele was associated with the anthropometric or biochemical response to the GENOMEX diet.

## 4. Discussion

This present study addressed the health effects of a regionalized GENOMEX diet and their relationship with the genetic profile of DRAG polymorphisms in subjects with metabolic risk factors for obesity-related CD. The main metabolic risk factors were consistent with the metabolic disturbances triggered by an increase in body fat, primarily visceral adipose tissue [42]. After 14-weeks with the GENOMEX diet, significant favorable changes in body composition were achieved and maintained throughout the complete dietary intervention. Improvement in body composition was entirely due to fat mass loss, as no changes in body water or muscle mass were observed.

Additionally, subjects were sedentary at baseline and were instructed to continue their usual pace of physical activity. Therefore, weight loss and maintenance during the study might also have been related to the macronutrient quality of the GENOMEX diet, which differed from that habitually consumed (Table 1). Dietary adherence was assessed and confirmed; however, after 14-weeks there was no additional significant loss or gain in anthropometric parameters. A meta-analysis conducted to compare intervention studies targeting weight loss using diet-only, exercise-only, or combined diet-and-exercise lifestyle intervention concluded that in the short-term (i.e., 3 to 6 months) diet-only and combined programs have similar effectiveness for weight loss, but in the long-term (i.e., 12 to 18 months) the most considerable weight loss arises from combined diet-and-exercise programs. Nonetheless, implementing exercise-only interventions were less effective than combined programs in both the short- and long-term [43]. Thus, the GENOMEX diet was effective for short-term weight loss in sedentary subjects, as they achieved an average loss of 6.6% of their initial weight as recommended by the Obesity Guidelines [44]. However, further studies are needed to assess its long-term efficacy for weight loss as well as in combination with exercise.

An amelioration of biochemical parameters was also observed in response to the regionalized GENOMEX diet, as it significantly reduced the frequency of individuals with IR, hypertriglyceridemia, and increased VLDL-c, as well as it improved fasting glucose and insulin values. Furthermore, although not statistically significant, it reduced the frequency of subjects with hypoalphalipoproteinemia. These improvements can be explained by the weight loss mainly of fat mass and the considerable reduction in waist circumference. Similarly, studies evaluating the metabolic effects of weight reduction have shown an improvement in both insulin sensitivity and lipid profile when a decrease in fat mass or at least an average weight loss of 5%–8% of baseline body weight is achieved [45,46]. Additionally, specific components or functional foods that made up most of the regionalized GENOMEX diet might have directly favored the improvement of these biochemical parameters. The nutritional properties and health benefits of foods such as beans, corn tortilla, amaranth, avocado, chia and pumpkin seeds, tomato, nopal (*Opuntia ficus-indica*), and quelites (*Chenopodium*) have been previously demonstrated [27,47]. For instance, the fiber-like properties of resistant starch in beans and corn tortilla have been shown to decrease postprandial glycemia/insulinemia, improve insulin sensitivity, and exert hypocholesterolemic actions [48]; the presence of fiber in nopal also provides antihyperglycemic and antihyperinsulinemic effects [49]; and the high content of essential fatty acids, phytochemicals, and fiber in amaranth, avocado and chia seeds exert antioxidant, anti-inflammatory and hypolipidemic effects [50,51,52]. These foods, including quelites, are also rich in folates, and a dietary folate intake lower than 300 µg/d has been associated with adiposity and insulin resistance in subjects with obesity [53]. Consistent with some of the metabolic effects of the regionalized GENOMEX diet are those of a controlled-feeding trial that assessed metabolic and inflammatory responses to a U.S. dietary pattern compared to a traditional Mexican diet in healthy women of Mexican descent [26]. The traditional Mexican diet improved insulin sensitivity (HOMA-IR), lowered serum concentrations of insulin-like growth factor-binding protein-3 (IGFBP-3), and tended to reduce concentrations of insulin-like growth factor-1 (IGF-1) under conditions of weight stability, while no difference in inflammatory response was revealed. However, that diet incorporated full-fat milk, which was one of the foods discarded in our study due to the high prevalence of the lactase non-persistence genotype in Mexico [15].

In particular, the regionalized GENOMEX diet was designed considering some DRAG polymorphisms [9] that in modern dietary and lifestyle environments have been associated with increased risk of unhealthy conditions [10,11,12,13,14,15,16]. Accordingly, we assumed that carriers of the adaptive alleles might require nutritional needs related to the main features of the pre-Hispanic diet (i.e., milk-free, scarce in animal meat, low in saturated fat and cholesterol, with low glycemic index and rich in antioxidants, fiber, and micronutrients such as folates due to the abundance of starchy foods, legumes, vegetables, and fruits). In this study, more than 48% of the subjects were lactase non-persistent (*LCT* -13910 CC), *MTHFR* CT/TT genotype carriers, and showed ≥6 copies of the *AMY1* gene. To a lesser extent, they presented the *ABCA1* 230C and *APOE* ε4 adaptive alleles. This genetic profile of study subjects from Guadalajara city was consistent with that exhibited by the general Mestizo population of West Mexico with an intermediate NA ancestry, including the Guadalajara population group previously analyzed [9]. Furthermore, *MTHFR* 677 CT+TT carriers showed a significant greater improvement in insulin sensitivity, while those having ≥ 6 *AMY1* copies improve this same feature, although not statistically significant. Notably, these individuals tended to be more insulin resistant at baseline compared to those without the respective adaptive variants. MTHFR is a key enzyme in the folate/homocysteine pathway. The MTHFR thermolabile variant encoded by the *MTHFR* 677 CT/TT genotypes leads to impaired homocysteine (Hcy) metabolism and elevated plasma Hcy levels, which has been associated with some metabolic diseases when coupled with insufficient dietary folate intake [10,11,54]. In particular, its association with accumulation of body and liver fat and increased risk of obesity have been explained by a possible mechanism in which elevated Hcy levels trigger an inflammatory process and increased production of cytokines, a metabolic feature that may cause insulin resistance, that later has been improved by a folate-rich diet [55]. Herein, the regionalized GENOMEX diet provided an adequate folate intake even higher than that usually consumed by individuals, and because the T allele was one of the most frequent among study subjects, it is plausible that the beneficial metabolic response was more evident compared to the other polymorphisms. Furthermore, subjects with ≥ 6 *AMY1* gene copies might also have benefited when exposed to this starch-rich low-glycemic diet, as previously observed in a study where compared to the low-amylase group (individuals with fewer *AMY1* gene copies and lower salivary amylase concentrations), the high-amylase/*AMY1* group had significantly lower post-prandial blood glucose, AUC and peak blood glucose values after starch intake [56]. However, contradictory results have emerged from studies evaluating the link between *AMY1* copy number and differential anthropometric and glycemic outcomes in response to dietary interventions [57]. Moreover, we did not find any correlation with the response to diet in the presence of the *ABCA1* 230C and *APOE* ε4 adaptive alleles; possibly due to the limited sample size, that among the study subjects few presented these alleles to show a remarkable response. Contrary to what was observed in a previous study amongst individuals with metabolic syndrome and the *ABCA1* R230C polymorphism, in which those having the 230C allele were more insulin resistant at baseline and showed a greater decrease in body weight and serum adiponectin in response to a dietary complement integrating Mexican foods [25].

Furthermore, the unmodified frequency of subjects with elevated values of TC, LDL-c, GGT, and ALT after the dietary intervention was an unpredictable finding. One explanation is that overall, HDL-c showed an increase, VLDL-c a decreased, but at the end of the study LDL-c showed an increase that led to an unmodified TC. Regarding the unmodified GGT and ALT values, some studies report a transient increase in liver enzymes during dietary-induced weight loss, primarily in women [58]. Although the causes remain unclear, transient liver injury due to hepatic lipid mobilization driven by body fat loss may be occurring.

This study is one of the few that addresses the preventive effects of a regionalized GENOMEX diet. The limitations are the lack of a comparative study group and the small sample size, which in this case allowed to observe significant changes in metabolic parameters but not to find significant gene-diet interactions for all DRAG polymorphisms. However, the effectiveness of this diet on weight loss and improvement of metabolic risk factors for obesity-related CD was proven, as well as the acceptance and adherence by the participants who reported pleasantness in consuming foods of Mexican origin that are regionally and economically accessible. Therefore, our findings suggest that this diet based on an ancestral genetic profile, regional food, and the food culture of the Mexican people can achieve healthy and preventive results by improving metabolic risk factors for obesity-related CD, such as excess of body weight and fat, IR, hypertriglyceridemia and increased VLDL-c values.

## 5. Conclusions

In conclusion, the regionalized GENOMEX diet significantly improved body composition and reduced the frequency of subjects with IR, hypertriglyceridemia and elevated VLDL-c; a more significant favorable effect on HOMA-IR and insulin was observed in *MTHFR* 677T adaptive allele carriers, but no other DRAG polymorphisms were associated with the clinical and biochemical changes in response to the diet. Studies evaluating the evolutionary history of the Mexican population are required to identify further genetic adaptations and the environment that triggered them in order to have a substantial scientific rationale for promoting early personalized preventive strategies.

## Figures and Tables

**Table 1:** Macro and micronutrient distribution of the GENOMEX diet and baseline dietary features of study subjects.

	GENOMEX Diet	Study Subjects (Baseline)	*p*	Reference Values [39]
Macronutrients				
Total energy (kcal)	1453.6 ± 113.0	2332.4 ± 853.0	<0.001	−
Protein (%)	20.1 ± 2.5	17.6 ± 4.2	<0.001	15–20
Total fat (%)	31.6 ± 5.1	31.3 ± 7.8	0.808	25–30
SFAs (%)	5.6 ± 3.9	9.0 ± 3.9	<0.001	<7
MUFAs (%)	11.5 ± 3.6	10.6 ± 4.0	0.208	10–15
PUFAs (%)	8.3 ± 2.5	5.1 ± 2.5	<0.001	7–10
Cholesterol (mg)	155.7 ± 105.3	300.4 ± 186.6	<0.001	<200
Carbohydrates (%)	52.7 ± 4.7	53.4 ± 9.0	0.642	50–55
Fiber (g/d)	32.0 ± 6.9	25.1 ± 11.7	0.001	25–38
Micronutrients				
Folates (µg/d of DFE)	301.0 ± 130.5	246.1 ± 164.6	0.022	300–600
Vitamin A (µg/d)	1342.9 ± 961.1	1198.5 ± 991.6	0.396	900
Vitamin C (mg/d)	269.3 ± 135.2	155.1 ± 117.5	<0.001	90
Vitamin E (mg/d)	6.1 ± 2.4	16.6 ± 73.5	0.392	15
Thiamin (mg/d)	1.3 ± 0.3	1.6 ± 0.6	0.007	1.1–1.2
Riboflavin (mg/d)	1.2 ± 0.3	1.6 ± 0.8	0.009	1.1–1.3
Niacin (mg/d)	14.2 ± 5.4	20.6 ± 8.8	<0.001	16
Pyridoxine (mg/d)	1.5 ± 0.4	1.7 ± 0.7	0.009	1.7
Cobalamin (µg/d)	2.1 ± 5.3	7.2 ± 20.5	0.138	2.4
Pantothenic acid (mg/d)	8.4 ± 21.6	6.1 ± 15.3	0.522	5
Calcium (mg/d)	1121.1 ± 428.9	1180.8 ± 588.3	0.454	1000
Iron (mg/d)	14.8 ± 2.9	17.6 ± 6.7	0.018	8–18
Sodium (mg/d)	1111.8 ± 481.3	1911.3 ± 1107.6	<0.001	1500
Potassium (mg/d)	2888.2 ± 614.3	3001.9 ± 1078.2	0.534	4700
Selenium (µg/d)	45.0 ± 18.4	52.8 ± 22.1	0.020	55
Phosphorus (mg/d)	721.7 ± 216.2	909.9 ± 419.0	0.011	700
Magnesium (mg/d)	367.1 ± 123.4	339.3 ± 175.2	0.359	310–420
Zinc (mg/d)	5.3 ± 1.7	10.8 ± 6.8	<0.001	8–11

SFAs, saturated fatty acids; MUFAs, monounsaturated fatty acids; PUFAs, polyunsaturated fatty acids; DFE, dietary folate equivalents.

**Table 2:** Genetic profile of DRAG polymorphisms in 37 study subjects.

*LCT*-13910 C>T		*MTHFR* 677 C>T		*ABCA1* R230C		*APOE* ε2, ε3, ε4		*AMY1* Copies	
CC	28 (75.7)	CC	19 (51.4)	RR	28 (75.7)	E2/E2	0 (0.0)	6.27 ± 2.9	
						E2/E3	2 (5.4)		
CT	8 (21.6)	CT	13 (35.1)	RC	9 (24.3)	E2/E4	0 (0.0)		
						E3/E3	28 (75.7)		
TT	1 (2.7)	TT	5 (13.5)	CC	0 (0.0)	E3/E4	7 (18.9)		
						E4/E4	0 (0.0)		
C	64 (86.5)	C	51 (68.9)	R	65 (87.8)	ε2	2 (2.7)	<6	18 (48.6)
T	10 (13.5)	T	23 (31.1)	C	9 (12.2)	ε3	65 (87.8)	≥6	19 (51.4)
						ε4	7 (9.5)		
HWE	0.371		0.137		0.352		0.588		---

*LCT*, lactase gene; *MTHFR*, methylenetetrahydrofolate reductase; *ABCA1*, ATP-binding cassette transporter A1; *APOE*, apolipoprotein E; *AMY1*, salivary amylase 1 gene. Genotypic and allele frequencies are expressed as number and percentage. *AMY1* copy number is expressed as mean ± standard deviation. HWE, Hardy–Weinberg equilibrium is reported as *p*-value of χ^2^-test.

**Table 3:** Dietary intervention effects on anthropometric and biochemical parameters (*n* = 33).

	Baseline	14 Weeks	24 Weeks	Total Change	*p* *	*p* **
Anthropometrics						
Weight (kg)	80.4 ± 18.5	74.5 ± 16.3	75.0 ± 16.6	5.3 ± 5.3	< 0.001	< 0.001
BMI (kg/m^2^)	30.0 ± 5.6	27.9 ± 5.1	28.0 ± 5.1	2.0 ± 1.9	< 0.001	< 0.001
WC (cm)	94.8 ± 14.7	88.5 ± 13.6	88.7 ± 14.1	5.9 ± 5.5	< 0.001	< 0.001
Body water (kg)	34.8 ± 8.4	35.2 ± 7.0	35.0 ± 6.8	−0.2 ± 3.8	0.794	0.780
Muscle mass (kg)	12.9 ± 2.6	12.9 ± 2.5	12.7 ± 2.6	0.3 ± 0.8	0.447	0.091
Fat mass (kg)	29.2 ± 11.4	23.9 ± 9.6	24.6 ± 10.1	4.6 ± 4.3	< 0.001	< 0.001
Body fat (%)	35.6 ± 7.0	31.3 ± 7.1	31.9 ± 7.9	3.7 ± 3.3	< 0.001	< 0.001
EBW (kg)	15.7 ± 10.4	9.8 ± 9.5	10.6 ± 10.3	5.3 ± 7.1	< 0.001	< 0.001
EFM (kg)	15.8 ± 10.4	10.1 ± 9.1	10.5 ± 10.2	5.3 ± 7.0	< 0.001	< 0.001
Normal weight ղ (%)	0 (0.0)	10 (30.3)	12 (36.4)	−12 (36.4)	−	< 0.001
Overweight ղ (%)	20 (60.6)	13 (39.4)	10 (30.3)	10 (30.3)	−	0.013
Obesity ղ (%)	13 (39.4)	8 (24.2)	11 (33.3)	2 (6.06)	−	0.609
Biochemicals						
Glucose (mg/dL)	89.0 ± 10.5	86.1 ± 11.4	84.3 ± 6.6	4.7 ± 8.7	0.326	0.004
Insulin (µU/mL)	15.0 ± 15.8	8.1 ± 4.0	8.9 ± 4.9	5.5 ± 14.2	< 0.001	0.002
HOMA-IR	3.6 ± 4.8	1.8 ± 0.8	1.9 ± 1.0	1.6 ± 4.3	0.001	0.002
TC (mg/dL)	185.2 ± 39.0	184.6 ± 38.0	187.5 ± 34.1	−3.4 ± 30.0	0.830	0.516
Triglycerides (mg/dL)	151.5 ± 88.3	115.9 ± 47.4	108.5 ± 44.7	43.0 ± 60.6	0.003	< 0.001
HDL-c (mg/dL)	41.2 ± 9.0	41.6 ± 6.8	43.2 ± 7.3	−0.8 ± 5.8	0.790	0.503
LDL-c (mg/dL)	112.0 ± 37.8	117.8 ± 32.2	124.8 ± 29.6	−14.3 ± 32.7	0.091	0.035
VLDL-c (mg/dL)	31.8 ± 21.5	23.5 ± 9.4	23.3 ± 11.3	9.0 ± 19.7	0.068	0.004
ALT (IU/L)	25.3 ± 15.9	21.8 ± 14.4	22.4 ± 14.7	2.6 ± 14.0	0.203	0.327
AST (IU/L)	21.4 ± 10.5	20.8 ± 16.2	20.1 ± 11.4	1.2 ± 6.8	0.033	0.295
GGT (IU/L)	27.5 ± 22.5	23.2 ± 16.6	25.2 ± 21.9	2.3 ± 10.7	0.060	0.110

BMI, body mass index; WC, waist circumference; EBW, excess body weight; EFM, excess fat mass; HOMA-IR, homeostasis model assessment of insulin resistance; TC, total cholesterol; HDL-c, high-density lipoprotein cholesterol; LDL-c, low-density lipoprotein cholesterol; VLDL-c, very low-density lipoprotein cholesterol; ALT, alanine aminotransferase; AST, aspartate aminotransferase; GGT, gamma-glutamyl-transferase. * *p*-value for changes between measurements, ** *p*-value for total change baseline vs. post-intervention.
